# Analysis of the quality, accuracy, and readability of patient information on polycystic ovarian syndrome (PCOS) on the internet available in English: a cross-sectional study

**DOI:** 10.1186/s12958-023-01100-x

**Published:** 2023-05-15

**Authors:** Helene Vågenes, Shelly Melissa Pranić

**Affiliations:** 1 University of Split School of Medicine, Šoltanska 2, Split, 21000 Croatia; 2 Cochrane Croatia, Šoltanska 2, Split, 21000 Croatia

**Keywords:** Polycystic ovarian syndrome, PCOS, Patient information, Health information online, Infertility, Obesity, Stein-Leventhal syndrome, Oligomennorhea, Anovulation, Hyperandrogenism

## Abstract

**Background:**

Online information about PCOS lacks reliability for patients seeking information about the disease. Thus, we aimed to perform an updated analysis of the quality, accuracy, and readability of patient information on PCOS available online.

**Methods:**

We conducted a cross-sectional study using the top five Google Trends search terms in English associated with PCOS, including “symptoms,” “treatment,” “test,” “pregnancy,” and “causes.” Five separate searches in Bing, Yahoo, and Google were performed to obtain the first 10 unique webpages for each term that was categorized as commercial, non-profit organization, scientific resources, or private foundation. We used the 16-item DISCERN with Likert-responses (minimum 1, maximum 5) where the total is 80 and lowest is 16, clarity with the 32-item EQIP, where responses of no = 0 and yes = 1 (minimum 0, maximum 32), and accuracy scores with 1 denoting poor and 5 completely accurate information; low scores of each corresponded to poorly reported information. We assessed readability with Flesch-Kincaid reading ease index, where higher scores correspond to reading ease, and lower grades correspond to easier readability with Flesch-Kincaid grade level, Gunning-Fog, Coleman-Liau index, automated readability index, New Dale-Chall Readability, and simple measure of gobbledygook. We additionally assessed word and sentence characteristics. We used Kruskal-Wallis test to compare scores according to webpage categories.

**Results:**

Out of 150 webpages, most were commercial (n = 85, 57%), followed by non-profit organizations (n = 44, 29%), scientific resources (n = 13, 9%) and private foundations (n = 6, 4%). Google webpages had higher median DISCERN score ([Md] = 47.0) than Bing ([Md] = 42.0) and Yahoo ([Md] = 43.0) webpages; *P* = 0.023. No difference in EQIP scores according to search engine was found (*P* = 0.524). Predominantly, webpages from private foundations had higher DISCERN and EQIP scores, although comparisons were not statistically significant (*P* = 0.456) and *P* = 0.653.). Accuracy and readability were similar across search engines and webpage categories (*P* = 0.915, range 5.0–5.0) and (*P* = 0.208, range 4.0–5.0).

**Conclusions:**

Quality and clarity of the data were fair according to search engine and category. Accuracy of information was high, showing that the public may encounter accurate information about PCOS. However, the readability of the information was high, reflecting a need for more readable resources about PCOS.

**Supplementary Information:**

The online version contains supplementary material available at 10.1186/s12958-023-01100-x.

## Background

Polycystic ovarian syndrome (PCOS) is a hormonal disorder occurring in women of reproductive age. Women with PCOS experience symptoms such as acne, hirsutism, amenorrhea, androgenic alopecia, and infertility together with obesity. PCOS causes symptoms that are challenging both physically and mentally for affected patients [1–3]. Many women with PCOS experience a lower quality of life due to psychological challenges like anxiety and depression [2, 3]. These symptoms together with the high prevalence of PCOS make it a topic frequently searched for on the internet by the public. Behboodi et al. showed in 2018 that several of these symptoms were widely reported as an important concern to women with PCOS, especially in adolescents [4]. Patients within this age group affected by this disorder predominantly use of internet as a major source of health information [5]. The information available online regarding PCOS is, however, of varying quality [1, 4, 6].

PCOS affects up to 15-20% of women of reproductive age, making it a common disorder in this patient group [2, 4]. Despite the high prevalence of PCOS in women, the diagnosis and differential diagnosis remain confusing. This is partly due to the lack of a specific test for providing the diagnosis, the prevalence of PCOS varying with diagnostic criteria, and diverse study settings and races [1]. Because clinical practice can be inconsistent regarding the assessment and management of PCOS, women internationally have highlighted delayed diagnosis and dissatisfaction with the care they are receiving [7]. There remains limited research synthesizing the broad clinical implications of PCOS, which would assist clinicians in the management of PCOS, and therefore benefit patient care [6]. The pathophysiological mechanisms of PCOS are complex and not fully understood. The etiology of PCOS has been suggested by multiple lines of evidence related with developmental, environmental, genetic, and epigenetic mechanisms [8]. It has become evident through recent years that race and ethnicity affect the clinical presentation of PCOS due to differences in genetic and environmental predispositions to endocrine and metabolic abnormalities [2]. Several studies have suggested that genetic factors have a central role in the etiology of PCOS [8]. The most conclusive evidence concerning the genetic predisposition for PCOS originated from research on genetic factors involving monozygotic and dizygotic twins. Monozygotic twins showed a higher concordance of PCOS symptoms compared to dizygotic twins [9].

Even though there are many aspects of the pathophysiology that remain unclear, it is widely accepted that hyperandrogenism plays a fundamental role. Excess androgen also impairs systemic metabolism via the brain by increasing adiposity and reducing insulin sensitivity [8]. Hyperinsulinemia promotes ovarian hyperandrogenism, which is present in 60–80% of women affected by PCOS [10]. However, the most common biochemical deviation in patients with PCOS is the elevation of circulating testosterone and androstenedione levels [8].

The major international diagnostic criteria currently proposed are the National Institutes of Health (NIH) standard, together with the Rotterdam criteria as well as the Androgen Excess Society (AES) criteria. The Rotterdam criteria suggested by the European society for human reproduction and Embryology/American Society for Reproductive Medicine is the diagnostic criteria used in most countries [1].

A major obstacle to effective healthcare for women affected by PCOS is the lack of recognition of the syndrome outside of gynecology and obstetrics. One of the central issues in increasing the awareness around PCOS beyond a subspecialty lies in the availability and familiarity with the required diagnostic procedures. An optimal management of PCOS would require a collaboration of a variety of healthcare professionals [11].

A recent 2022 study by Ismayilova et al. found through interviews of women in Canada that there is a perceived lack of knowledge on PCOS from both physicians and patients, highlighting the need for improvement in knowledge and awareness of PCOS in primary health care. This study also shed light on the need for more resources, and further PCOS research to be funded and conducted. Moreover, the authors reported participants’ desire for credible doctor-provided information to be made available, especially age-specific support together with mental health support groups [12].

These facts highlight how PCOS is a public health problem for which insufficient information may be available for people who mostly seek health information on the internet. Accessing the internet is now a fundamental part of the lives more than 5 billion people around the world who have access, and regulations set forth by the World Health Organization and European Commission acknowledge the significant influence on citizens’ understanding and use of information from the internet and digital technologies [13][14]. Accordingly, previous investigations have shown that populations who have the ability to use the internet to seek health-related information and critically appraise the information have optimal healthcare use and communicate better with providers [15–17]. Of the few studies that have performed evaluations of the quality of publicly available information regarding PCOS on the internet, they describe the dearth of high quality information for the lay public. Namely, Saroja & Chandrashekar assessed the quality of information on the symptoms and diagnosis of PCOS found on the internet according to a non-standardized evaluation [18]. Mousiolis and colleagues reported low quality information on the symptoms, diagnosis, and treatment of PCOS based on Health on the Net Foundation (HON) criteria for the top 15 internet webpages concerning PCOS in 2012 [19]. A 2018 study conducted by Chiu et al. determined the low quality and readability, along with the lack of user-friendliness of the webpage content on PCOS from mostly commercial sources [5]. However, none of these studies used standardized tools to assess the quality of the information on PCOS. Thus, an updated study using standardized instruments to assess the quality of publicly available information on the internet on PCOS is warranted [5].

Given that both the quality and clarity of information concerning PCOS available for patients on the internet has not yet been assessed with standardized tools, we aimed to determine the quality and clarity of information on PCOS available on the internet using the validated DISCERN tool [20], as well as the EQIP tool [21]. We also assessed the reading grade level with eight standardized tests [22]. Lastly, we assessed the accuracy of symptoms described in the web pages about PCOS through comparisons to recent systematic reviews [2, 23–25].

## Materials and methods

### Electronic searches

We chose the top five keywords directly related to PCOS from Google Trends on February 28, 2022 to conduct subsequently our searches in the separate search engines. The keywords included [1] polycystic ovary syndrome symptoms, [2] polycystic ovary syndrome treatment, [3] polycystic ovary syndrome causes, [4] polycystic ovary syndrome pregnancy, and [5] polycystic ovary syndrome test [26]. One investigator performed the keyword search. We used Google^®^, Bing^®^, and Yahoo^®^ search engines for separate searches not limited to any specific geographical region to search for webpages using the abovementioned keywords describing PCOS information in English. Google Chrome version 99 (99.0. 4844.88) was used for all the searches.

### Inclusion and exclusion criteria

We included webpages with information describing information about PCOS intended for patients or the lay public (newspaper websites, government and academic institutions, health center or hospital websites, or non-profit institutions), that had text with more than 30 sentences or 100 words long, and had identifiable domains (e.g., “.com”, .edu, “.gov”, “.info”, “.net”, “.biz”) to allow for their categorization. Webpages were categorized as commercial (.com), scientific resources (.edu and .gov), private foundations/advocacy (.health, .info, and .net), and non-profit organizations (.org) [27]. We excluded webpages that required a subscription, were videos, scientific journal articles, were intended for health care professionals, or were inaccessible.

One investigator (HV) copied and pasted the text of unique, non-duplicate webpages from each search from March to May 2022. Another investigator (SMP) checked the eligibility. Any disagreement about the categorization of the webpages was discussed until consensus was reached without the involvement of a third author. Two investigators rated the quality, clarity, and accuracy of the PCOS information with the DISCERN and EQIP tools in a 10% random sample of the webpages (5 from each search engine).

Inter-rater reliability was high for DISCERN items (kappa range 0.826 to 1.00). We resolved through consensus discussion the differences in our interpretation of item 9 “description of how each treatment works”, which had the lowest kappa in any single category of the DISCERN items (0.83, 95% confidence interval [CI] 0.77–0.89), before the full data extraction by HV. Inter-rater reliability was high for EQIP items (kappa range 0.83 to 1.00). We resolved through consensus discussion the differences in our interpretation of item 26 “use of generic names”, which had the lowest kappa in any single category of the EQIP items (0.83, 95% confidence interval (CI) 0.77–0.88) before the full data extraction by HV.

Inter-rater reliability was also high for accuracy of symptoms with a kappa range 0.891 (95% CI 0.84–0.94) to 1.00. We resolved through consensus discussion the differences in our interpretation before the full data extraction by HV.

### Data collection

We extracted the top 10 webpages from each of the three databases from which we performed five separate searches using the keywords chosen from Google Trends [26]. The web history log and cookies were deleted between each keyword and webpage search to avoid the influence of previous searches. The text and images were copied and pasted into Google Word documents marked with the date of extraction. We assessed the quality of the PCOS information using the DISCERN tool and the clarity with the EQIP tool. Due to the presence of figures and other images needed to rate the webpages that might have otherwise become altered when pasted to the Word document, we scored the webpages in live format directly, given that the date of the latest review or update was prior to the initial text extraction date. When this was not possible, a copy of the extracted text was used. We stipulated a maximum two-clicks per webpage to access the information included in the assessment.

### Evaluation instruments

#### The DISCERN tool

The 16-item DISCERN tool utilizes four criteria to assess the authorship, attribution, currency of information, and ownership of a publication (website owner and conflict of interest of health information in written form. The tool contains Likert scores of 1 (no), 2, 3 (partially), 4, and 5 (yes) for items 1–15 to judge the presence of [1] clear aims, [2] information that addresses the stated aims, [3] relevant or realistic treatment information, [4] references to the sources used as evidence, [5] dates of the main sources of information, [6] a bias assessment, [7] suggestions for further reading or additional sources of information, [8] acknowledgement of gaps in knowledge, [9] the effectiveness of each treatment, [10] benefits of each treatment, [11] a description of the risks of each treatment, [12] descriptions of disease progression in the absence of treatment, [13] adverse events and the impact on the overall quality of life, [14] description of treatment choice options, [15] suggestions to discuss the health information on the website with family or health practitioners. To facilitate rating the webpages, we separated the intermediate ratings to 2 (somewhat low), 3 (moderate), 4 (somewhat high), while the lowest and highest ratings remained 1 (low or not available) and 5 (high). Item 16 is a summary score that addresses overall quality, denoted as 1 (low), 2, 3 (moderate), 4, and 5 (high). The minimum DISCERN score is 16, while the maximum score is 80. The quality of the information was classified according to the median score as “excellent” (63 to 80), “good” (51 to 62), “fair” (39 to 50), “poor” (28 to 38), or “very poor” (≤ 27).

#### The EQIP tool

We used the modified 36-item EQIP tool to assess the clarity of the PCOS information. There are 18 items related to content, 6 items for the identification of information, and 12 items regarding the structure of the information. For each item, we recorded yes or no responses to indicate the presence or absence of information and used not applicable (N/A) if the item in question was not relevant for a particular webpage. We excluded Item 27 from the EQIP because this item describes the “use of short sentences (< 15 words on average)” which was already automatically assessed in a separate readability analysis that we performed (described in the next section). Therefore, the maximum total EQIP score was 35 in the present study. Webpages with an EQIP score greater than 22.0, which corresponds to the 75th percentile, were deemed as high-scoring webpages. Low-scoring webpages were those with an EQIP score less than or equal to 22.0 [21].

### Readability

Readability was analyzed using the online readability calculator at readable.io by directly pasting the webpage text from and including the title to the last sentence into the readability calculator [1]. We reported the Flesch-Kincaid Reading Ease scored from 0 to 100, where lower scores indicate difficult to understand text and higher scores indicate easier reading. Lower grade levels correspond to easier readability with the Flesch-Kincaid grade (FKG) level (ranges from grade 0 to 18 [college graduate] the Gunning-Fog (GF) score (ranges from grade 0 to 20 [college graduate]. The Coleman-Liau index (CLI) and automated readability index (ARI), ranging from 5 to 22 (college graduate), the New Dale-Chall Readability (NDCR) [ranges from grade level 4 to college graduate], and simple measure of gobbledygook (SMOG) [ranges from grade level 3 to college graduate] scores correspond to the years of education needed to understand written material. We additionally collected word count, syllables per word, words with more than two syllables, words per sentence, and sentence count.

### Accuracy of symptoms

We assessed the accurate and inaccurate statements of the symptoms of PCOS on the webpages using systematic reviews published from 2019 to 2022 [2, 23–25]. We based the accuracy of the PCOS information on the proportion of total accurate statements on the symptoms in the webpage text compared to the total number of statements about symptoms. Symptoms not accepted as accurate symptoms according to the systematic reviews included enlarged clitoris, headache, sleep apnea, sleep problems, pelvic pain, eating disorders, sexual dysfunction, oily skin, deeper voice, decreased breast size, mood changes, insomnia, fatigue, increased appetite, hypertension, swollen belly, endometrial hyperplasia, hidradenitis suppurativa, fatty liver, recurrent miscarriage, hyperkeratosis, inappropriate male features, and behavioral changes together with urinary and fecal incontinence. We assessed the accuracy of symptoms by counting the number of accurate listed symptoms in the website text and counting the total number of statements or words describing symptoms on a webpage. With the goal of producing one accuracy score for each website, the total number of accurate symptom descriptions was divided by the total number of statements or words describing symptoms. Scores were based on the proportion of accurate data ranging from 1 (lowest) to 5 (highest) [28, 29].

A score of 0 was assigned when the webpage did not list any symptoms. A score of 1 represented less than 25% agreement with evidence-based information, a score of 2 represented 26-50% agreement with evidence-based information, a score of 3 represented 51-75% agreement with evidence-based information, a score of 4 represented 76-99% agreement, and a score of 5 denoted 100% agreement with evidence-based sources on PCOS symptoms [28].

### Statistical analysis

The DISCERN, EQIP, readability, and accuracy scores were treated as continuous variables. The EQIP responses were treated as dichotomous (rated as a 1 for yes or 0 for no) categorical variables, where each item scored as 1 contributed to the total score for each webpage. We used the Kolmogorov-Smirnoff test to determine the distribution of the numerical data and used non-parametric tests for non-normally distributed numerical variables. We reported descriptive data as n (%), median (Md), and interquartile range (IQR). Our analysis of webpage quality, clarity, and accuracy involved the assessment of the interrater reliability with Cohen’s kappa for agreement along with 95% confidence intervals (CI). The Kruskal-Wallis test was used to determine whether the DISCERN, EQIP, readability, and accuracy scores differed between search engine or webpage category. Dunn-Bonferroni post-hoc analysis was used for the Kruskal-Wallis test to determine in which category differences existed. The statistical significance was set at P < 0.05 for all the comparisons. MedCalc version 9.1.2 (MedCalc software bv) and IBM SPSS Statistics for Windows, versions 22.0 (IBM Corp., Armonk, N.Y., USA) were used for all analyses.

## Results

This study included 150 webpages describing information about PCOS from the searches performed in Google, Bing, and Yahoo. Table 1 shows the overall characteristics of the webpages.

**Table 1:** Producer type and readability scores of polycystic ovary syndrome (PCOS) webpages

Characteristics		Total
**Webpage producer type**		
Commercial, n (%)*		87 (58%)
Non-profit organizations, n (%)		44 (29%)
Scientific resources, n (%)		13 (9%)
Private foundation, n (%)		6 (4%)
Total, n (%)		150 (100%)
**Readability scores**		**Median (IQR**†**)**
Flesch Reading Ease		48.3 (42.0–54.0)
Flesch-Kincaid Grade Level		10.0 (9.0–11.0)
Gunning Fog Score		12.0 (11.0–13.0)
Coleman Liau Index		12.0 (11.0–13.0)
Simple measure of gobbledygook (SMOG) Index		12.0 (11.0–13.0)
Automated Readability Index		9.0 (8.0–10.0)
Spache Readability Score		4.0 (4.0–5.0)
Dale-Chall Readability Score		7.0 (6.0–7.0)
Word count		1020.0 (730.0-1534.0)
Syllables per word		2.0 (2.0–2.0)
Words with more than four syllables		14.0 (9.0–23.0)
Words per sentence		13.0 (11.0–15.0)
Sentence count		79.0 (58.0-116.0)

*Designated as having .com domain names.

†Interquartile range (IQR).

Out of the 150 webpages collected for this study, 87 (58%) were from commercial producers. While 44 (29%) webpages were from non-profit organizations, 13 (9%) were from scientific resources which include academic and government webpages. The remaining 6 (4%) were from private foundations. Most of the webpages originated from the USA (n = 89, 59%), followed by the UK (n = 20, 13%) and India (n = 16, 11%) [Additional File 1].

### DISCERN scores

The overall median DISCERN score for the webpages was 44.0 (IQR 36.0–51.0). Google webpages describing PCOS had a higher median DISCERN score of 47.0 (IQR 39.0–55.0) compared to webpages from Bing (median 42.0, IQR 35.0–48.0) or Yahoo (median 43.0, IQR 35.0–48.0); *P* = 0.023. Post-hoc pairwise comparisons showed differences between Google vs. Bing and Yahoo (*P* = 0.010 and *P* = 0.034, respectively). The overall DISCERN score for organization webpages was 47.0 (IQR 37.27-51.0). For scientific resources the score was 45.0 (IQR 38.0–51.0), while private foundations had a score of 43.5 (IQR 34.5–54.0). Commercial webpages had a median of 43.0 (IQR 35.0–51.0). The median DISCERN score was highest for non-profit organization webpages 47.0 (IQR 37.27-51.0), compared to the lowest of commercial webpages 43.0 (IQR 35.0–51.0), but this difference was non-significant (*P* = 0.456). The quality of the information was classified according to the median score as “excellent” (63 to 80), “good” (51 to 62), “fair” (39 to 50), “poor” (28 to 38), or “very poor” (< 27). Overall, there were 39 (26.0%) webpages rated as good or excellent, 58 (39.0%) were rated as fair, and 53 (35.0%) were rated as poor or very poor. Webpages having a score greater than 44, which we considered as the minimum score of quality ratings, were found for 59.1% of webpages from non-profit organizations and 53.8% for scientific resources, compared to 41.4% of commercial webpages.

### EQIP scores

The median EQIP score was 20 (IQR 18.0–22.0). Webpages with an EQIP score of greater than 22.0, which corresponds to the 75th percentile, were deemed as high-scoring webpages, while we deemed low-scoring webpages as those with an EQIP score less than or equal to 22.0. A high score was achieved by 36 (24%) of webpages and the remaining 114 (76%) achieved a low score. The lowest score achieved was 5 by one webpage obtained from a commercial webpage from Yahoo, whereas the highest score of 29 was obtained by a non-profit organization website from Google.

There was no significant difference in the EQIP scores according to Google (median 20.5, IQR 18.0-23-0), Bing (median 20.0, IQR 17.0–22.0), or Yahoo (median 20.0, IQR 18.0–22.0); *P* = 0.524.

According to webpage producer type, the median EQIP score for commercial webpages was 20.00 (IQR 18.0–23.0). For non-profit organization webpages, the EQIP score was 20.00 (IQR 18.0–22.0). Scientific resources had a median EQIP score of 20.00 (IQR 18.5–21.0), while private foundations had a median of 22.00 (IQR 19.25–23.50). Webpages from private foundations had a higher median EQIP score of 22.00 (IQR 19.25–23.50); *P* = 0.653, but the comparisons by producer type were not significant.

### Accuracy scores

According to webpage producer type the accuracy score was 5.00 (IQR 4.0–5.0) for both commercial and non-profit organization webpages. Scientific resources had a median of 5 (IQR 4.5-5.0) as well, while private foundations had a median of 4.00 (IQR 0.0–5.0) *P =* 0.208.

We found no significant difference in the accuracy score of webpages across the three search engines regardless of the producer type (5.0 [IQR 4–5]); *P* = 0.915.

### Readability scores

The reading grade level of the webpages ranged from 7 to 12, where most webpages were written at the tenth-grade reading level (Table 2).

**Table 2:** Readability scores and text characteristics of PCOS webpages in Google, Bing, and Yahoo search engines

Readability scores median (IQR)*	Google (n = 50)		Bing (n = 50)	Yahoo (n = 50)	*P*†
Flesch-Kincaid reading ease	49 (41–56)	48 (42–54)		48 (42–54)	0.787
Flesch-Kincaid grade level	10 (8–11)	10 (9–11)		10 (9–11)	0.787
Gunning-Fog score	12 (11–13)	12 (11–13)		12 (10–13)	0.787
Coleman-Liau	12 (11–13)	12 (11–13)		12 (11–13)	0.571
SMOG^‡^	12 (11–13)	12 (11–13)		12 (11–13)	0.923
Automated readability index	9 (7–11)	9 (12–13)		9 (11–13)	0.571
Dale-Chall Readability	7 (6–7)	7 (7–7)		7 (6–7)	0.197
Word count	1132 (807–1922)	923 (648–1444)		891 (681–1518)	0.353
Syllables per word	2 (2–2)	2 (2–2)		2 (2–2)	0.580
Words with more than four syllables	15 (8–27)	14 (10–23)		14 (10–23)	0.830
Words per sentence	13 (11–15)	13 (11–15)		13 (11–14)	0.987
Sentence count	89 (65–147)	76 (47–108)		77 (51–113)	0.383

*IQR – interquartile range.

†Kruskal-Wallis test with a P-value set at 0.05.

‡Simple measure of gobbledygook (SMOG) index.

Google had a higher median Flesch-Kincaid reading ease of 49 (IQR 41–56), compared to a median of 48 (IQR 42–54) for Bing and a median of 48 (IQR 42–56) for Yahoo, but the comparison did not reach statistical significance (*P* = 0.787). The median Flesh-Kincaid grade level did not differ across the three search engines. The readability scores by producer type are shown in Table 3.

**Table 3:** Readability of webpages describing PCOS information by producer type

Readability scores median (IQR)*	Commercial	Organization (.org)	Scientific resources	Private foundation	*P* ^†^	
Flesch-Kincaid reading ease	48 (41–52)	49 (42–56)	53 (47–60)	49 (40–57)	0.248	
Flesch-Kincaid grade level	10 (8–11)	10 (8–11)	9 (8–10)	10 (10–12)	0.132	
Gunning-Fog score	12 (11–13)	12 (10–13)	11 (19 − 13)	13.05 (12–14)	0.174	
Coleman-Liau	12 (11–13)	12 (11–13)	11 (10–12)	11.87 (11–13)	0.437	
SMOG^‡^	12 (12–13)	12 (11–13)	12 (11–13)	13 (12–14)	0.151	
Automated readability index	9 (8–11)	9 (8–10)	8 (7–9)	10 (9–11)	0.161	
Dale-Chall Readability	7 (6–7)	7 (6–7)	7 (6–7)	7 (6–7)	0.561	
Word count	1020 (748–1577)	983 (657–1449)	897 (779–1212)	1095 (711–1210)	0.953	
Syllables per word	1.70 (1.70–1.80)	1.70 (1.60–1.80)	1.70 (1.60–1.70)	1.70 (1.60–1.80)	0.391	
Words with more than four syllables	15 (10–24)	14 (7–24)	17 (7–20)	11 (6–17)	0.529	
Words per sentence	13 (11–15)	12 (11–14)	13 (11–14)	15 (13–17)	0.092	
Sentence count	78 (60–117)	86 (53–130)	76 (58–116)	62 (48–90)	0.842	

*IQR – interquartile range.

^†^Kruskal-Wallis test with a P-value set at 0.05.

^‡^Simple measure of gobbledygook (SMOG) index.

Websites from scientific resources showed a higher median Flesch-Kincaid reading ease, of 53 (47–60), compared to commercial websites with a median of 48 (42–52), private foundations with a median of 49 (40–57), and non-profit organization websites 49 (42–56), but a statistically significant difference was not found (*P =* 0.248). The Flesch-Kincaid grade level was lower for scientific resources 9 [8–10] compared to commercial and non-profit organization websites with a median of 10 [8–11] and private foundations with a median of 10 [10–12], but these comparisons did not reach statistical significance (*P =* 0.132).

## Discussion

In this cross-sectional study on the quality, readability, and accuracy of information on PCOS, we found that the information was accurate, but quality and readability were not high. Most of the webpages had a commercial domain, which is in concordance with previous similar studies that showed a tendency for health information to originate from mostly commercially produced webpages [30, 31]. Similar to findings by Chiu et al., the quality of commercial webpages was low as shown by the EQIP, and we additionally found that commercial webpages had low DISCERN scores. To this end, other studies on PCOS and various diseases have found that commercial webpages contain information of low quality or clarity [5, 12, 18, 32–35]. The lowest EQIP score was achieved by a commercial webpage found in Yahoo, while the highest score was obtained by a non-profit organization website by Google, which suggests a higher clarity from non-commercial webpages. The median EQIP for private foundation webpages was higher as compared to commercial, non-profit organization and scientific resources, but no significant difference was established.

According to search engine, the DISCERN median score was higher for Google as compared to Bing and Yahoo, showing a significant difference in quality, suggesting a possible advantage in using Google as a search engine. The study also did not find a significant difference in clarity between the three search engines: Google, Bing and Yahoo.

The accuracy of symptoms was found to be high across the search engines and producers [2, 23–25]. Our study found that only three websites did not list any symptoms in their content, in which two were produced by private foundations and one by a non-profit organization, respectively. Similar research assessing accuracy and readability of pancreatic cancer also showed high levels of accuracy, especially from government webpages. In contrast to the present study, previous studies based their accuracy on an expert panel or consultations with patients and clinicians informed by professional guidelines [28, 35]. As many patients self-diagnose based on information found on the internet, this data suggests that the majority of symptoms on PCOS available online in English are accurate [21].

All webpages had grade levels that were above the fifth grade, which exceeds the recommendation by the joint commission for written educational materials for patients. The median readability score for commercial, private foundations and organization webpages were at a median tenth-grade reading level, while the score for scientific resource webpages were at a median ninth-grade reading level. Our findings corroborate with the reading difficulty of information on webpages intended for the general public found by previous investigations, that is, having a readability at or above the eighth grade. The joint commission recommends that all patient education materials should be written at or below the fifth-grade reading level to meet the health literacy needs of the public, suggesting a need for readability levels to be improved [5, 36, 37]. It should also be noted that PCOS is a disorder affecting adolescent girls as well, who usually read at a sixth, seventh, or eighth-grade level. Moreover, since the internet has become an increasingly ubiquitous source for health information, the low readability of information could affect patients’ critical appraisal of information to inform a constructive relationship between them and healthcare workers [21, 38, 39]. Since more medical journals include patients’ viewpoints in the writing of scientific articles, the materials on PCOS should be clear and easy to read.

Current initiatives seek to empower laypeople to be aware of potentially inaccurate or unreliable content that may arise from unregulated sources [30]. The objectives set forth by the US Office of Disease Prevention and Health Promotion since 2010 intended to promote US citizens’ understanding of online health information to prevent harm from wrong or inaccurate information [40–42]. Further in the EU, the European Union Directorate-General for Communications Networks, Content, and Technology aim to improve EU citizens’ health literacy, that is empower EU citizens to better appraise, use, and access relevant and evidence-based information on the internet to guide their health care decisions[43]. The World Health Organization also has plans to increase the health literacy of the world’s population as a part of their 2030 Agenda for Sustainable Development [44]. Considering the varying readability of health information, using plain language, substituting complex medical terms with simpler terms, and shortening sentences is of utmost importance for laypeople’s understanding of PCOS information found on the internet.

The present study used a robust methodology with validated tools to assess the quality, clarity, and readability of internet-based information about PCOS that has been similarly used in many other assessments of the quality of online information for other reproductive and pregnancy-related diseases and patient-related concerns [35, 45–47]. We additionally reduced potential bias due to subjective assessments of quality and clarity through establishing Cohen’s Κ reliability between the investigators. Thus, the methodology attests to the relevance of our results to help clinicians in practice dispel low quality sources and empower their patients to seek high quality ones surrounding PCOS on the internet. Our study adds to the literature robust and updated evidence for multiple stakeholders.

Despite our findings being in accordance with previous research, our study presented some limitations. The webpage searches were not exhaustive, utilizing only five search terms selected by Google Trends. The identification of search terms with Google Trends only provides the most used search phrases by the wider public, possibly not truly predicting the search patterns of individuals seeking information on PCOS online [21]. The study is limited by a small sample size of 150 webpages with an unequal distribution across producer types.

Additionally, only the top ten webpages from each search term were investigated. The study results were also limited to web pages in English, researchers from other countries may evaluate online information on PCOS in other languages. As such, the data from this study may differ from conclusions drawn on patient information in other languages. The study also did not make any differentiations or grouping regarding the origin country of the information analyzed. We did not assess the construct validity of the DISCERN tool with the modified ratings. However, the expanded ratings were still within the range of the original ratings, reducing the possibility that we somehow overestimated the quality of the webpages.

Further, we did not include video-based material in our study, limiting our study to text. We have utilized the EQIP tool to analyze websites containing information regarding PCOS although the tool was not originally created for this specific purpose, therefore making it a possible limitation [21]. Validated quality indicators are needed to help improve the quality and clarity of PCOS information available online as most of them achieved low scores overall, which highlights similar research findings showing a need to improve online resources providing health information [5, 12, 32]. Additionally, we did not assess the quality of the systematic reviews used to assess the accuracy of the symptoms reported about PCOS in this study.

Health care workers should educate themselves about the quality, clarity, and readability characteristics of PCOS webpages. In this way, health care professionals should strive to educate their patients on how to navigate and interpret high-quality online-based information that may guide patients’ health-related decision-making. Finally, it is important to note that the findings of this study represent a snapshot of a point in time from when the search was performed. However, while search engine results may change over time, we consider the findings to be representative of the information available to patients on PCOS online in English.

## Conclusion

Analogous to previous investigations on the quality of health-related information available on the internet, the majority of webpages describing information about PCOS had suboptimal quality and clarity according to the DISCERN tool and EQIP tools. The reading grade level of the information on PCOS was higher than the recommended fifth-grade reading level regardless of producer type, funder, or search engine. Our findings highlight the need for increased patient and provider awareness of PCOS content that comprise quality and comprehendible information to facilitate decision-making. High-quality PCOS-related online information available in English within the recommended readability level is lacking and there is a need for high-quality, user-friendly PCOS patient information online.

## Electronic supplementary material

Below is the link to the electronic supplementary material.

Additional File 1. Description of the quality and readability characteristics of the PCOS webpages according to countries of webpage origin (.xls file extension).

## Data Availability

The datasets supporting the conclusions of this article are available in the Open Science Framework repository, [https://osf.io/6kzv9/].

## Abbreviations

PCOS: Polycystic ovarian syndrome
